# TIGIT/CD155 axis mediates resistance to immunotherapy in patients with melanoma with the inflamed tumor microenvironment

**DOI:** 10.1136/jitc-2021-003134

**Published:** 2021-05-10

**Authors:** Shusuke Kawashima, Takashi Inozume, Masahito Kawazu, Toshihide Ueno, Joji Nagasaki, Etsuko Tanji, Akiko Honobe, Takehiro Ohnuma, Tatsuyoshi Kawamura, Yoshiyasu Umeda, Yasuhiro Nakamura, Tomonori Kawasaki, Yukiko Kiniwa, Osamu Yamasaki, Satoshi Fukushima, Yuzuru Ikehara, Hiroyuki Mano, Yutaka Suzuki, Hiroyoshi Nishikawa, Hiroyuki Matsue, Yosuke Togashi

**Affiliations:** 1 Research Institute, Chiba Cancer Center, Chiba, Japan; 2 Department of Dermatology, Chiba University Graduate School of Medicine, Chiba, Japan; 3 Department of Dermatology, University of Yamanashi, Chuo, Japan; 4 Division of Cellular Signaling, National Cancer Center Research Institute, Chuo-ku, Japan; 5 Department of Dermatology and Plastic Surgery, Faculty of Life Sciences, Kumamoto University, Kumamoto, Japan; 6 Department of Skin Oncology/Dermatology, Saitama Medical University International Medical Center, Hidaka, Japan; 7 Department of Pathology, Saitama Medical University International Medical Center, Hidaka, Japan; 8 Department of Dermatology, Shinshu University School of Medicine, Matsumoto, Japan; 9 Department of Dermatology, Okayama University Graduate School of Medicine, Dentistry and Pharmaceutical Sciences, Okayama, Japan; 10 Department of Molecular and Tumor Pathology, Chiba University Graduate School of Medicine, Chiba, Japan; 11 Department of Computational Biology and Medical Sciences, Graduate School of Frontier Sciences, The University of Tokyo, Kashiwa, Japan; 12 Division of Cancer Immunology, Research Institute/Exploratory Oncology Research and Clinical Trial Center (EPOC), National Cancer Center, Chuo-ku/Kashiwa, Japan; 13 Department of Immunology, Nagoya University Graduate School of Medicine, Nagoya, Japan; 14 Department of Tumor Microenvironment, Okayama University Graduate School of Medicine, Dentistry and Pharmaceutical Sciences, Okayama, Japan

**Keywords:** costimulatory and inhibitory t-cell receptors, drug therapy, combination, immunotherapy, melanoma, tumor microenvironment

## Abstract

**Background:**

Patients with cancer benefit from treatment with immune checkpoint inhibitors (ICIs), and those with an inflamed tumor microenvironment (TME) and/or high tumor mutation burden (TMB), particularly, tend to respond to ICIs; however, some patients fail, whereas others acquire resistance after initial response despite the inflamed TME and/or high TMB. We assessed the detailed biological mechanisms of resistance to ICIs such as programmed death 1 and/or cytotoxic T-lymphocyte-associated protein 4 blockade therapies using clinical samples.

**Methods:**

We established four pairs of autologous tumor cell lines and tumor-infiltrating lymphocytes (TILs) from patients with melanoma treated with ICIs. These tumor cell lines and TILs were subjected to comprehensive analyses and in vitro functional assays. We assessed tumor volume and TILs in vivo mouse models to validate identified mechanism. Furthermore, we analyzed additional clinical samples from another large melanoma cohort.

**Results:**

Two patients were super-responders, and the others acquired resistance: the first patient had a non-inflamed TME and acquired resistance due to the loss of the beta-2 microglobulin gene, and the other acquired resistance despite having inflamed TME and extremely high TMB which are reportedly predictive biomarkers. Tumor cell line and paired TIL analyses showed high CD155, TIGIT ligand, and TIGIT expression in the tumor cell line and tumor-infiltrating T cells, respectively. TIGIT blockade or CD155-deletion activated T cells in a functional assay using an autologous cell line and paired TILs from this patient. CD155 expression increased in surviving tumor cells after coculturing with TILs from a responder, which suppressed TIGIT^+^ T-cell activation. Consistently, TIGIT blockade or CD155-deletion could aid in overcoming resistance to ICIs in vivo mouse models. In clinical samples, CD155 was related to resistance to ICIs in patients with melanoma with an inflamed TME, including both primary and acquired resistance.

**Conclusions:**

The TIGIT/CD155 axis mediates resistance to ICIs in patients with melanoma with an inflamed TME, promoting the development of TIGIT blockade therapies in such patients with cancer.

## Background

Cancer acquires mechanisms to escape the immune system during development and progression.^1 2^ One such mechanism involves the induction of inhibitory molecules, such as programmed death 1 (PD-1)/PD-1 ligands and cytotoxic T-lymphocyte-associated protein 4 (CTLA-4).^1–4^ Immune checkpoint inhibitors (ICIs) against these molecules improve the outcome of various types of cancer including melanoma and lung cancer.^5–7^ However, their efficacy as monotherapy is unsatisfactory, with a response rate of less than 50%.

PD-1, which interacts with PD-1 ligands, is primarily expressed following the activation of T cells and suppresses T cell function, falling T cells into a dysfunctional exhausted state.^8 9^ ICIs reinvigorate dysfunctional exhausted T cells, leading to tumor regression.^10–14^ Thus, the inflamed tumor microenvironment (TME) (ie, highly CD8^+^ T-cell-infiltrated TME) is a predictive biomarker of ICIs.^11–14^ Another well-known predictive biomarker is the tumor mutation burden (TMB).^13–16^ Since neoantigens derived from somatic mutations induce strong immune responses as non-self-antigens, patients with high TMB, which reflect a high number of neoantigens, reportedly respond favorably to ICIs.^10 15 16^ However, the efficacy of ICIs remains inadequate even when these biomarkers are present.^13 14^ Indeed, several controversies have been reported.^13 14 17^ In particular, some patients with inflamed TME and/or high TMB fail to respond to ICIs in clinical settings.

It is important to elucidate the resistance mechanisms to identify biomarkers and increase the efficacy of the treatment.^18 19^ Patients resistant to ICIs can be divided into two groups: those who primarily fail to respond (primary resistance) and those who initially respond but eventually develop disease progression (acquired resistance).^18 19^ There are several known mechanisms such as non-inflamed TME, lack of sufficient or suitable neoantigens, loss-of-function genomic alterations in interferon (IFN)-γ signaling pathways, loss of beta-2 microglobulin (B2M) subunit of major histocompatibility complex class I (MHC-I), and upregulation of other inhibitory checkpoint molecules.^18–23^ However, the mechanisms of both primary and acquired resistance to ICIs have not been fully understood, which may be due to the complexity of human cancer and also of the immune system. In addition, heterogeneous human clinical samples appear to be significantly different from homogeneous animal models. Thus, further research using human clinical samples is warranted.

Here, we established four pairs of tumor cell lines and cultured tumor-infiltrating lymphocytes (TILs) from four patients with melanoma who received ICIs. Two were super-responders, and the remaining two acquired resistance after the initial response. Pathological analyses, whole-exome sequencing (WES), and RNA sequencing (RNA-seq) showed that a patient acquired resistance despite an inflamed TME and extremely high TMB. Using comprehensive analyses and functional assays, we identified that the TIGIT/CD155 axis, one of the inhibitory molecules,^24–26^ contributed to the acquired resistance. In another melanoma cohort, CD155 was related to resistance to ICIs in patients with inflamed TME, including both primary and acquired resistance. We propose that the TIGIT/CD155 axis mediates resistance to ICIs in patients with cancer with inflamed TME, including acquired resistance, and that TIGIT blockade therapies should be developed for such patients.

## Materials and methods

### Patients and samples

Four patients with melanoma who underwent surgical resection at Yamanashi University Hospital from 2017 to 2019, were enrolled in this study to establish autologous tumor cell lines and cultured TILs (online supplemental table S1). All participants provided written informed consent. Tumor specimens collected from the patients were processed as previously described.^23^ Briefly, surgically resected tumors were enzymatically digested with collagenase, hyaluronidase, and deoxyribonuclease (Sigma-Aldrich, St. Louis, Missouri, USA) in RPMI1640 (Thermo Fisher Scientific, Waltham, Massachusetts, USA) at room temperature. After filtration and separation according to density gradient, the digested tumors were used.

10.1136/jitc-2021-003134.supp1 Supplementary data

In addition to these patients, 144 patients with melanoma treated with anti-PD-1 monoclonal antibody (mAb) and/or anti-CTLA-4 mAb whose formalin-fixed, paraffin-embedded (FFPE) tissues were available at Yamanashi University Hospital, Chiba University Hospital, Shinshu University Hospital, Saitama Medical University International Medical Center, Okayama University Hospital, and Kumamoto University Hospital from 2014 to 2020 were enrolled in this study as another cohort (online supplemental tables 2 and 3). Patients who received ICIs as second-line therapies received BRAF/MEK inhibitors before ICIs, and their samples were obtained before treatment with BRAF/MEK inhibitors. If available, we also obtained both pretreatment and post-treatment paired samples from patients who developed resistance after treatment. Patients’ clinical information was obtained from their medical records.

### In vitro expansion of TILs

TILs were cultured and expanded as previously described.^23^ In brief, melanoma tumor digests were initiated in RPMI1640 supplemented with 10% human AB serum, antibiotics, and recombinant human interleukin 2 (rhIL-2: 6000 IU/mL, PeproTech, Cranbury, New Jersey USA) in a humidified 37°C incubator with 5% CO_2_. Half of the media was aspirated from the wells and replaced with fresh complete medium and rhIL-2 every 2–3 days.

### Cell lines

To establish tumor cell lines, 1×10^7^ digested tumor cells were cultured in RPMI1640, containing 10% fetal bovine serum (FBS; Cytiva, Tokyo, Japan), penicillin, streptomycin, and amphotericin B (Thermo Fisher Scientific). Tumor cells were passaged at approximately 80%–90% confluence and used when free of fibroblasts and proliferating beyond the 10th passage. The MEL01 cell line was generated from a previously reported patient with melanoma who acquired resistance after an initial response to PD-1 blockade.^23^ This cell line lost the B2M gene and had no MHC class I expression.^23^ Both MEL02 and MEL03 cell lines were generated from melanoma super-responders to PD-1 blockade before the initiation of therapy. The MEL04 cell line was generated from a patient with melanoma who acquired resistance to anti-PD-1 and anti-CTLA-4 mAbs after initial response to anti-PD-1 mAb (online supplemental table S1 and figure S1).

**Figure 1:**
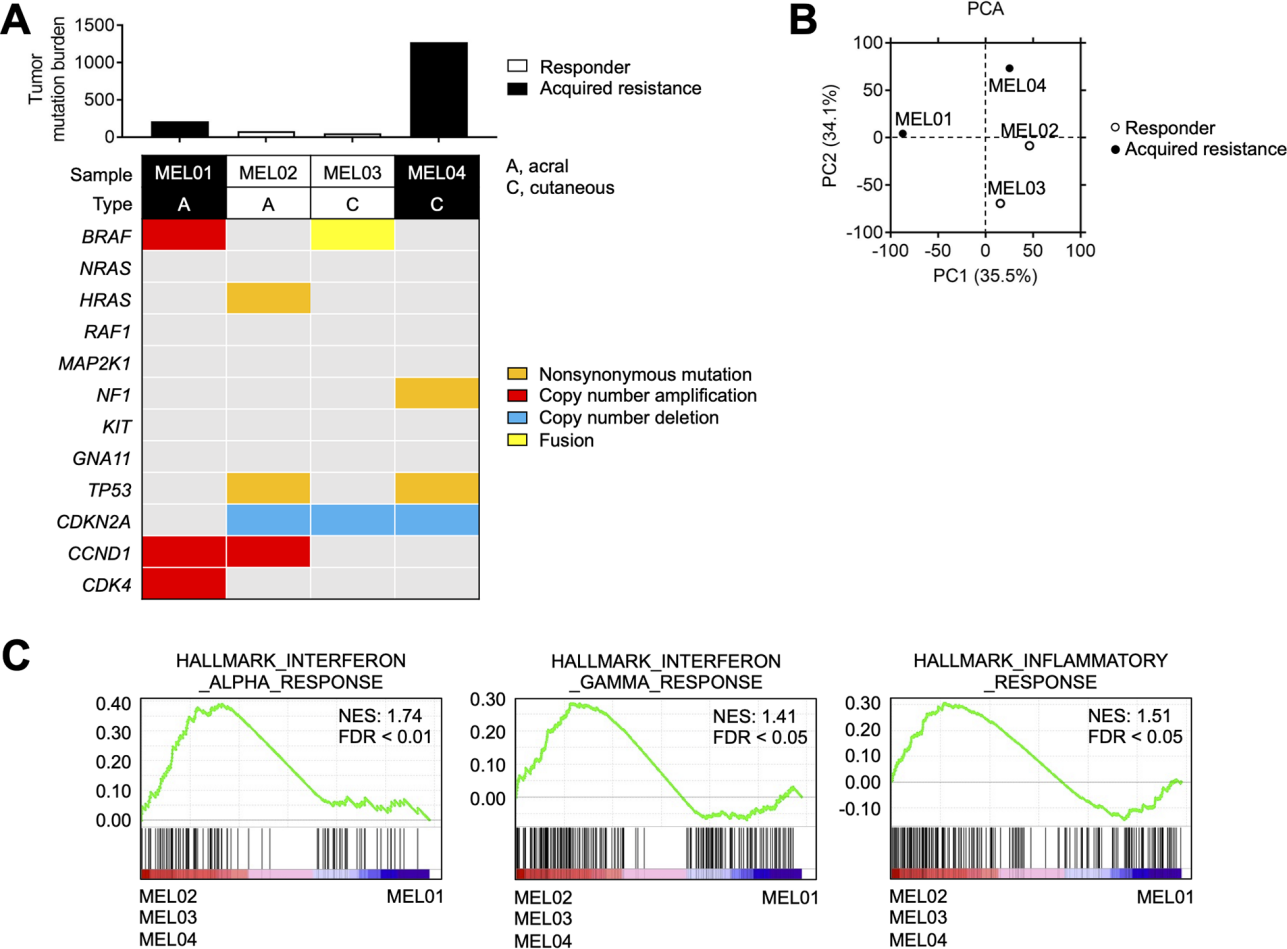
MEL04 involving inflamed tumor microenvironment and extremely high TMB. (A) TMB and representative driver gene alterations in four melanoma cell lines. Whole-exome sequencing was performed for each cell line. The number of non-synonymous mutations and representative driver gene alterations of melanoma are shown. (B) PCA in four melanoma cell lines. We conducted a PCA based on variable gene expression (top 10,000 SD) from the RNA-sequencing data. (C) Immune-related gene signatures from gene set enrichment analysis in MEL02, 03, and 04 compared with MEL01. Interferon-α, interferon-γ, and inflammatory response related gene signatures from the MSigDB database are shown. PCA, principal component analysis; TMB, tumor mutation burden.

B16F10 (mouse melanoma) and EMT6 (mouse breast cancer) cell lines were purchased from ATCC (Manassas, Virginia, USA) and were maintained in the RPMI1640 medium supplemented with 10% FBS. All tumor cells were used after confirming that they were *Mycoplasma* (−) after *Mycoplasma* testing with the PCR Mycoplasma Detection Kit (TaKaRa, Shiga, Japan) according to the manufacturer’s instructions.

### WES

Genomic DNA was isolated from each melanoma cell line and paired peripheral blood using a QIAamp DNA Mini Kit (QIAGEN, Hulsterweg, Netherlands) and enriched for exonic fragments using the SureSelect Human All Exon Kit v6 (Agilent Technologies, Santa Clara, California, USA). Massive parallel sequencing of the isolated fragments was performed with the HiSeq3000 instrument (Illumina, San Diego, California, USA) using the paired-end method. WES reads that masked nucleotides with a quality value of <20 were independently aligned to the human reference genome (hg38) using BWA (http://bio-bwa.sourceforge.net/) and Bowtie2 (http://bowtie-bio.sourceforge.net/bowtie2/index.shtml). Both somatic synonymous and non-synonymous mutations were called using our in-house caller and two publicly available mutation callers: Genome Analysis Toolkit (https://gatk.broadinstitute.org/hc/en-us) MuTect2 and VarScan2 (http://varscan.sourceforge.net/). Mutations were discarded if any of the following criteria were met: the total read number was <20, the variant allele frequency (VAF) in the tumor samples was <0.05, the mutant read number in the germline control samples was >2, the mutation occurred in only one strand of the genome, or the variant was present in the normal human genome in either the 1000 Genomes Project data set (https://www.internationalgenome.org/) or our in-house database. Gene mutations were annotated using SnpEff (https://pcingola.github.io/SnpEff/). Copy number status was analyzed using our in-house pipeline, which determines the logR ratio (LRR) as follows: (1) Single nucleotide polymorphism (SNP) positions in the 1000 Genomes Project database that were in a homozygous state (VAF≤0.05, or ≥0.95) or a heterozygous state (VAF=0.4–0.6) in the genomes of respective normal samples were selected; (2) normal and tumor read depths at the selected position were adjusted based on the G+C percentage of a 100 bp window flanking the position; (3) the LRR was calculated as $log_{2}\frac{t_{i}}{n_{i}}$, where $n_{i}$ and $t_{i}$ are the normal and tumor-adjusted depths at position *i*, respectively, and (4) each representative LRR was determined as the median of a moving window (1 Mb) centered at position i.

### RNA-seq and expression analysis

Total RNA was extracted from each melanoma cell line using the RNeasy Mini Kit (Qiagen). RNA-seq libraries were prepared using the NEBNext Ultra RNA Library Prep Kit (New England BioLabs, Ipswich, Massachusetts, USA), in which complementary DNA (cDNA) was prepared from polyA-selected RNA. The prepared RNA-seq libraries were subjected to next-generation sequencing from both ends (paired-end reads). The expression level of each gene was calculated using DESeq2 (http://bioconductor.org/packages/release/bioc/html/DESeq2.html) with variance-stabilizing transformation (VST).

### Principal component analysis and gene set enrichment analysis

Principal component analysis (PCA) based on the top 10,000 variable genes was performed using R software (R Foundation for statistical computing). Enriched pathways were determined using the gene set enrichment analysis (GSEA) tool available on the Broad Institute website. Hallmark gene sets were downloaded from the MSigDB database.^27^

### Quantitative real-time reverse transcription polymerase chain reaction (qRT-PCR)

Total RNA was reverse-transcribed to cDNA using PrimeScript RT Master Mix (TaKaRa), and real-time PCR was performed using TB Green Premix Ex Taq II (TaKaRa) according to the manufacturer’s instructions. *GAPDH* was used as internal control. The experiments were performed in triplicate. The primers used are listed in online supplemental table S4.

### Human *PVR-*deletion or mouse *pvr*-deletion using CRISPR/Cas9 technology

Human *PVR-*knockout (KO) MEL04, mouse *pvr*-KO B16F10, and EMT6 cell lines were generated using CRISPR/Cas9 technology. Briefly, a targeting guide RNA (gRNA) sequence (human, 5ʹ-CGTTTGGACTCCGAATAGCT-3ʹ; mouse, 5ʹ-TCAAATAACCTGGATGAAGA-3ʹ) was used to edit the genomic locus. The gRNA and Cas9 protein (Thermo Fisher Scientific) were transfected into MEL04, B16F10, or EMT6 cells using lipofectamine CRISPRMAX (Thermo Fisher Scientific). The expression of CD155 was evaluated using flow cytometry in triplicate.

### IFN-γ release functional assay

Cultured autologous tumor cell lines (10^5^ cells/well) were used as stimulator cells. TILs (10^5^ cells/well) were added to the tumor cells, and were incubated for 24 hours. The supernatants were assayed with ELISA for IFN-γ (Thermo Fisher Scientific). Anti-TIGIT mAb (MBSA43, Thermo Fisher Scientific), anti-LAG-3 mAb (11C3C65, BioLegend, San Diego, California, USA), anti-TIM-3 mAb (F38-2F2, BioLegend), and anti-MHC-I mAb (W6/32, BioLegend) were added at a concentration of 10 µg/mL. In vitro experiments were performed in triplicates.

### TIGIT expression analyses by cultured T cells

Autologous tumor cells were cocultured with paired TILs for 48 hours, and subsequently, TIGIT expression was analyzed with flow cytometry. In addition, TILs or peripheral blood mononuclear cells (PBMCs) from healthy donors were stimulated by anti-CD3 mAb (OKT3, Thermo Fisher Scientific) at indicated concentrations and anti-CD28 mAb (CD28.8, Thermo Fisher Scientific) at 0.5 µg/mL. Forty-eight hours later, TIGIT expression was analyzed with flow cytometry. In vitro experiments were performed in triplicates.

### In vivo animal study

Female C57BL/6J mice (6–8 weeks old) were purchased from CLEA Japan (Tokyo, Japan). C57BL/6J-Prkdc<scid>/Rbrc mice (B6 SCID; RBRC01346) were provided by RIKEN BRC (Tsukuba, Japan) through the National BioResource Project of the MEXT/AMED, Japan. Cells (1×10^6^) were injected subcutaneously, and the tumor volume was monitored two times a week. The mean values of the long and short diameters were used to generate tumor growth curves. Mice were grouped when the tumor volume reached approximately 100 mm^3^, and ICIs (anti-PD-1 mAb, 200 µg/mouse; anti-CTLA-4 mAb, 100 µg/mouse; anti-TIGIT mAb, 100 µg/mouse) were administered intraperitoneally three times every 3 days thereafter. Tumors were harvested 7 days after treatment initiation to collect TILs for evaluation. Rat anti-mouse PD-1 mAb (RMP1-14) were purchased from BioXcell (West Lebanon, New Hampshire, USA). Mouse anti-mouse CTLA-4 mAb (9D9-IgG2a) and mouse anti-mouse TIGIT mAb (1B4) were purchased from absolute antibody (Oxford, UK). In vivo experiments were performed at least two times. All mice were maintained under specific pathogen-free conditions at the animal facility of the Institute of Biophysics. Mouse experiments were approved by the Animal Committee for Animal Experimentation of the Chiba Cancer Center. All experiments met the US Public Health Service Policy on the Humane Care and Use of Laboratory Animals.

### Flow cytometry analyses

Flow cytometry assays were performed as previously described.^28^ Briefly, cells were washed with phosphate-buffered saline containing 2% FBS and stained with surface antibodies. Intracellular staining was performed with specific antibodies and the FOXP3/Transcription Factor Staining Buffer set (Thermo Fisher Scientific) according to the manufacturer’s instructions. For intracellular cytokine staining, the GolgiPlug reagent (BD Biosciences) was added for the last 4 hours of culture. Samples were assessed with BD Canto II or BD FACSVerse and FlowJo software (BD Biosciences). The staining antibodies were diluted according to the manufacturer’s instructions. The antibodies used in flow cytometry are listed in online supplemental table S5.

### Immunohistochemistry

Sections of FFPE tissue (3 µm) were dried, dewaxed, and rehydrated. Immunohistochemical staining for both CD8 and CD155 was performed automatically using the Ventana XT system BenchMark (Ventana Medical Systems, Tucson, Arizona, USA), as previously described.^29^ Briefly, the tissue sections were automatically treated with an antigen retrieval solution (Ventana) and heated on a slide heater at 100°C for 30 min. Endogenous peroxidase activity was quenched by immersion in 3% hydrogen peroxide for 4 min. The sections were then incubated with anti-CD8 mAb (CB/144B, Dako, 1/100 dilution) or anti-CD155 mAb (D3G7H, Cell Signaling Technology, 1/500 dilution) for 30 min at 37°C. Detection was performed using the LSAB Ventana Iview DAB detection system (Ventana) according to the manufacturer’s instructions. The sections were counterstained with hematoxylin and blindly reviewed by pathologists (TKawas and YI). Membrane expression of CD155 in tumor cells was assessed as previously reported.^30^ Intratumoral CD8^+^ T cells were counted: five fields 0.25 mm^2^ were randomly selected and counted for each slide as previously reported.^31^ For each patient, the mean value of the counts in five areas was used for the statistical analysis. We set cut-offs for CD8 count and CD155 score from the receiver operating characteristic (ROC) curves using 6-month progression-free survival (PFS).

### Statistical analyses

GraphPad Prism V.8 (GraphPad Software), JMP Pro V.16.0 (SAS Institute), or R V.4.0.2, was used for statistical analyses. Patients’ characteristics were compared between two groups using the Fisher’s exact test. The relationships of continuous variables between or among groups were compared using the t-test or one-way analysis of variance (ANOVA), respectively. The relationships between tumor volume curves were compared using two-way ANOVA. For multiple testing, Bonferroni corrections were employed. PFS and overall survival (OS) were defined as the time from the initiation of first ICIs until the first observation of disease progression or death from any cause and the time from the initiation of first ICIs until death from any cause, respectively. PFS and OS were analyzed using a Kaplan-Meier method and compared among groups using a log-rank test. A Cox proportional hazards model was used for the univariate and multivariate analyses to estimate HRs and 95% CIs. P values <0.05 were considered statistically significant.

## Results

### A patient with melanoma acquired resistance to ICIs despite the inflamed TME and extremely high TMB

A man in his early 70s (MEL04) with cutaneous melanoma received anti-PD-1 mAb monotherapy as first-line therapy and achieved partial response (online supplemental figure S1A). Thirty months later, however, he acquired resistance and had brain and subcutaneous metastases. Anti-CTLA-4 mAb was added but he failed to respond (online supplemental figure S1A). The subcutaneous lesion was resected. Pathological analyses of the resected lesion revealed that tumor-infiltrating CD8^+^ T cells increased after the treatment, which is different from that in a previously reported patient with melanoma who acquired resistance due to loss of the B2M gene (MEL01) (online supplemental figure S1B and table S1).^23^ Accordingly, MHC-I was highly expressed in MEL04 tumor cells (online supplemental figure S1C).

Next, we performed WES and RNA-seq using four melanoma cell lines from two responders (MEL02 and MEL03) and two non-responders (acquired resistance) (MEL01 and MEL04) to ICIs (online supplemental table S1). Representative driver oncogene alterations and TMB are summarized in figure 1A, which show that MEL04 had an extremely high TMB. PCA using RNA-seq data showed that the MEL04 cell line was different from MEL01, although both were acquired-resistance cell lines. In contrast, the MEL04 cell line was similar to the responders’ MEL02 and MEL03 cell lines (figure 1B). Next, we performed GSEA to identify differences between MEL01 and MEL02, MEL03, and MEL04, resulting in the enrichment of immune-related gene signatures (figure 1C). Altogether, MEL04 acquired resistance to ICIs despite the inflamed TME and extremely high TMB.

### TIGIT/CD155 axis mediates resistance to immunotherapy

**Figure 2:**
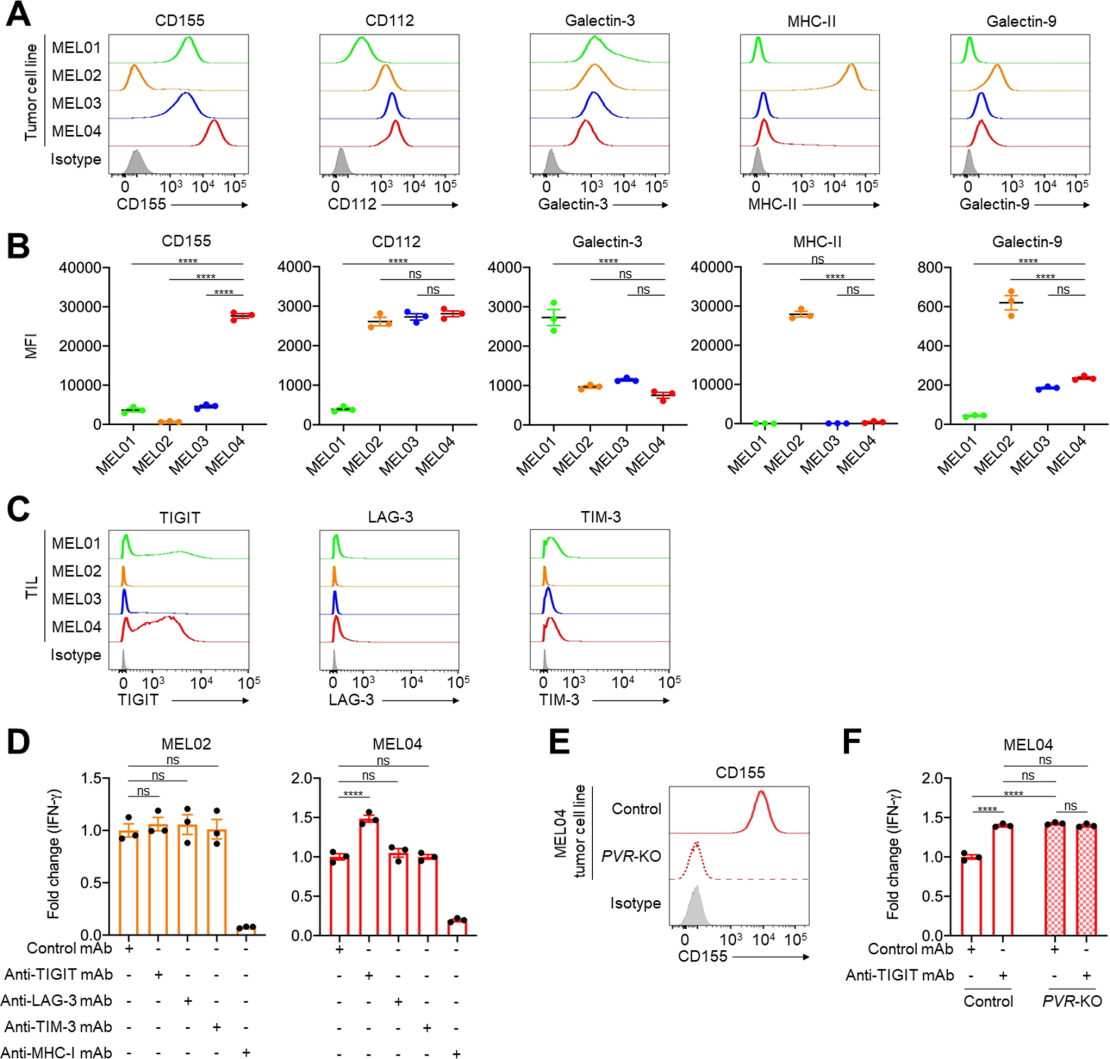
The TIGIT/CD155 axis mediates resistance to immunotherapy from functional assays using clinical samples. (A and B) Expression of TIGIT, LAG-3, and TIM-3 ligands in tumor cells. We selected CD155 and CD112 as TIGIT ligands, galectin-3, and MHC class II (MHC-II) as LAG-3 ligands, and galectin-9 as a TIM-3 ligand. Representative flow cytometry staining (A) and summaries (B) are shown. (C) TIGIT, LAG-3, and TIM-3 expression in tumor-infiltrating T cells. Surgically resected tumors were digested, and the digested products before culture and expansion were subsequently analyzed with flow cytometry. (D) IFN-γ release assay of MEL02 (left) and MEL04 (right). Autologous tumor cells and paired TILs were cocultured with or without each immune checkpoint inhibitors for 24 hours. Supernatants were analyzed with ELISA for IFN-γ. Anti-MHC-I mAb was used for a negative control. Fold changes to samples with control mAb are shown. (E) CD155 expression in MEL04 tumor cells. The PVR gene was knocked out in the MEL04 cell line using CRISPR/Cas9 technology (*PVR*-KO), and the created cell line was analyzed by flow cytometry. Representative flow cytometry staining from triplicated experiments is shown. (F) IFN-γ release assay of MEL04. In vitro experiments were performed as described in (D). All in vitro experiments were performed in triplicate, and one-way analysis of variance with Bonferroni corrections were used in (B), (D), and (F) for statistical analyses. The means and SEM are shown. ****, p<0.0001; ns, not significant; IFN-γ, interferon-γ; MFI, mean fluorescent intensity; TIL, tumor-infiltrating lymphocyte.

In general, both inflamed TME and high TMB are well-known predictive biomarkers for ICIs, which is inconsistent with MEL04.^10–16^ These findings prompted us to analyze immune suppressive factors, especially co-inhibitory checkpoint molecules other than PD-1 or CTLA-4. Among the co-inhibitory checkpoint molecules from RNA-seq, the MEL04 cell line showed high expression of *LGALS9*, *PVR*, and *NECTIN2* (encoding Galectin9, CD155, and CD112, respectively) compared with the other cell lines (online supplemental figure S1D). We validated the results using qRT-PCR and flow cytometry, which showed that CD155, a TIGIT ligand, was highly expressed only in MEL04 cells (figure 2A, B, and online supplemental figure S1E). Next, we analyzed immune checkpoint molecules expressed in tumor-infiltrating T cells before expansion in these four patients. Accordingly, TIGIT was particularly highly expressed in tumor-infiltrating T cells of MEL04 (figure 2C). These findings suggest that MEL04 could confer resistance to ICIs due to the TIGIT/CD155 axis.

To validate the role of the TIGIT/CD155 axis in the resistance mechanism of MEL04, we performed an IFN-γ release functional assay using pairs of autologous tumor cell lines and TILs from the same patients. A pair from MEL02 was used as a control since the cell line and TILs had low CD155 and low TIGIT expression, respectively. IFN-γ released by coculturing with the MEL02 cell line and TILs was not increased by TIGIT blockade (figure 2D). In contrast, IFN-γ release was increased by TIGIT blockade but not by the others (LAG-3 and TIM-3) in MEL04 (figure 2D). These results are consistent with high TIGIT but low LAG-3 and TIM-3 expression in tumor-infiltrating T cells in MEL04 (figure 2C). Next, we created the *PVR*-KO MEL04 cell line using CRISPR/Cas9 technology and performed a similar functional assay (figure 2E). Consistently, *PVR*-deletion increased IFN-γ release, and the addition of the TIGIT blockade had no effect (figure 2F). These findings indicate that the TIGIT/CD155 axis mediates resistance to ICIs in CD155-expressing tumors with tumor-infiltrating TIGIT^+^ T cells.

### Tumor cells acquire resistance to immunotherapy through the TIGIT/CD155 axis

**Figure 3:**
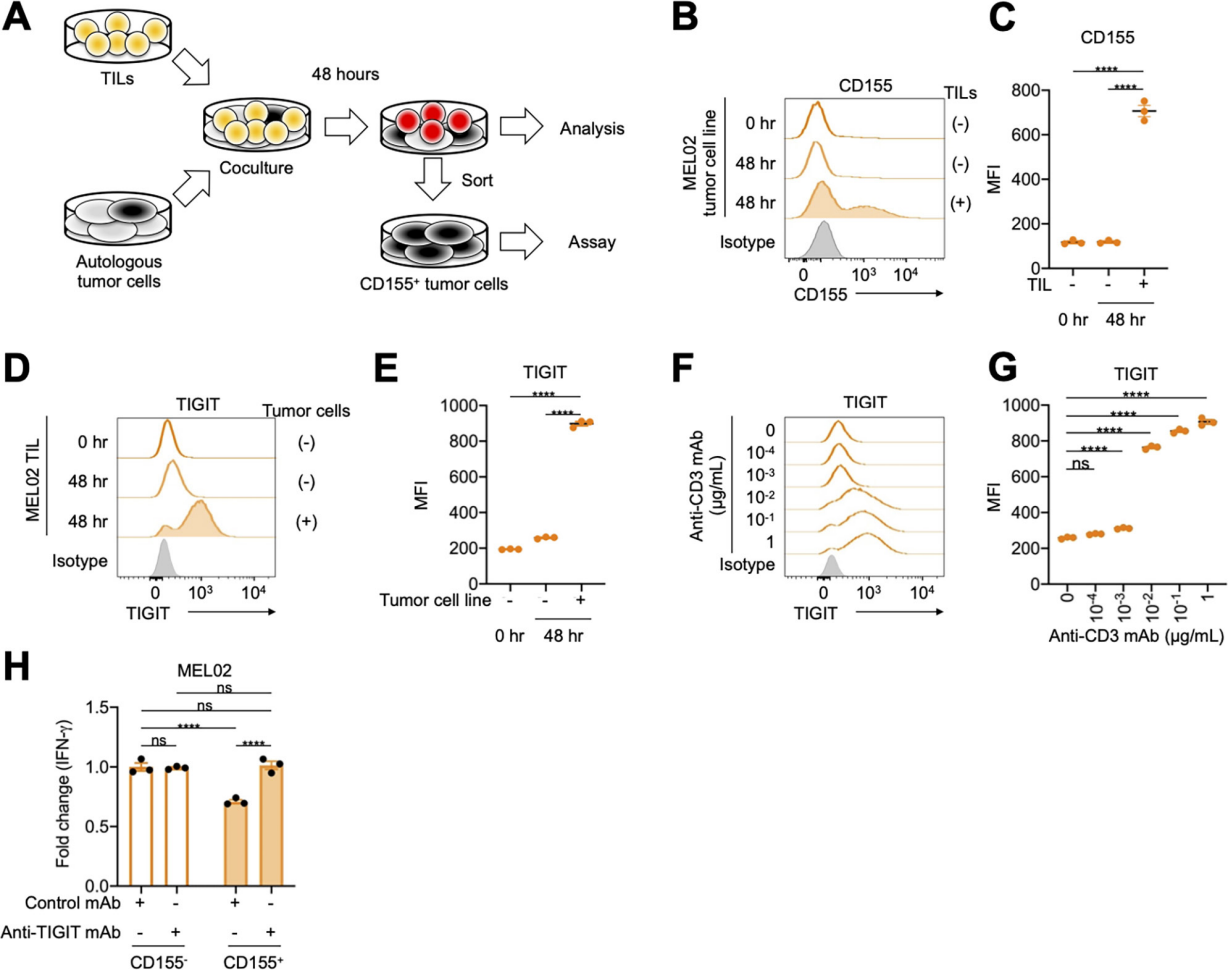
The TIGIT/CD155 axis induces acquired resistance to immunotherapy. (A) Graphical experimental schema of the in vitro acquired resistance model. Autologous tumor cells were cocultured with paired TILs for 48 hours, and both were subsequently analyzed with flow cytometry. In addition, CD155-expressing tumor cells were sorted and subjected to functional assay. (B–E) CD155 expression in MEL02 tumor cells and TIGIT expression in tumor-infiltrating T cells from MEL02. Expression was analyzed 48 hours after coculture as described in (A). Representative flow cytometry staining (B, tumor cells; D, tumor-infiltrating T cells) and summaries of MFI (C, tumor cells; E, tumor-infiltrating T cells) are shown. (F and G) TIGIT expression in tumor-infiltrating T cells from MEL02. MEL02 TILs were stimulated by anti-CD3 mAb with indicated concentrations and anti-CD28 mAb for 48 hours and were subsequently analyzed with flow cytometry. Representative flow cytometry staining (F) and summary of MFI (G) are shown. (H) IFN-γ release assay. CD155^-^ or CD155^+^ autologous tumor cells and paired TILs from MEL02 were cocultured with or without anti-TIGIT mAb for 24 hours. Supernatants were analyzed with ELISA for IFN-γ. Fold changes to CD155^−^ tumor cells with control mAb are shown. All in vitro experiments were performed in triplicate, and one-way analysis of variance with Bonferroni corrections were used in (C), (E), (G), and (H) for statistical analyses. The means and SEM are shown. ****, p<0.0001; ns, not significant; hr, hour; IFN-γ, interferon γ; MFI, mean fluorescent intensity; TILs, tumor-infiltrating lymphocytes.

We established an acquired resistance model using the pair of MEL02 cell line and TILs (figure 3A). Surviving tumor cells and TILs after coculture had higher CD155 and TIGIT expression than the controls (figure 3B–E). Similarly, anti-CD3 mAb increased TIGIT expression in CD8^+^ T cells in a dose-dependent manner (figure 3F, G, and online supplemental figure S2), indicating that T-cell receptor (TCR) signaling pathways increase TIGIT expression. In addition, TIGIT is reportedly considered a chronically stimulated exhausted T cell marker.^8 9^ To imitate chronic stimulation, we also cocultured for 1 month exchanging TILs every week, showing that surviving tumor cells had high CD155 expression (online supplemental figure S3). Accordingly, surviving CD155-expressing MEL02 tumor cells suppressed T-cell activation, which was canceled by TIGIT blockade (figure 3H). Since PD-1 reportedly inhibits TCR signaling pathways, PD-1 blockade activates these pathways.^32^ Altogether, TCR signaling pathways, which can be activated by PD-1 blockade, increase TIGIT expression in effector T cells, and TIGIT^+^ effector T cells are suppressed by CD155 expressed in tumor cells, resulting in the survival of CD155-expressing tumor cells and acquired resistance.

### TIGIT/CD155 blockade overcomes resistance to ICIs in in vivo mouse models

**Figure 4:**
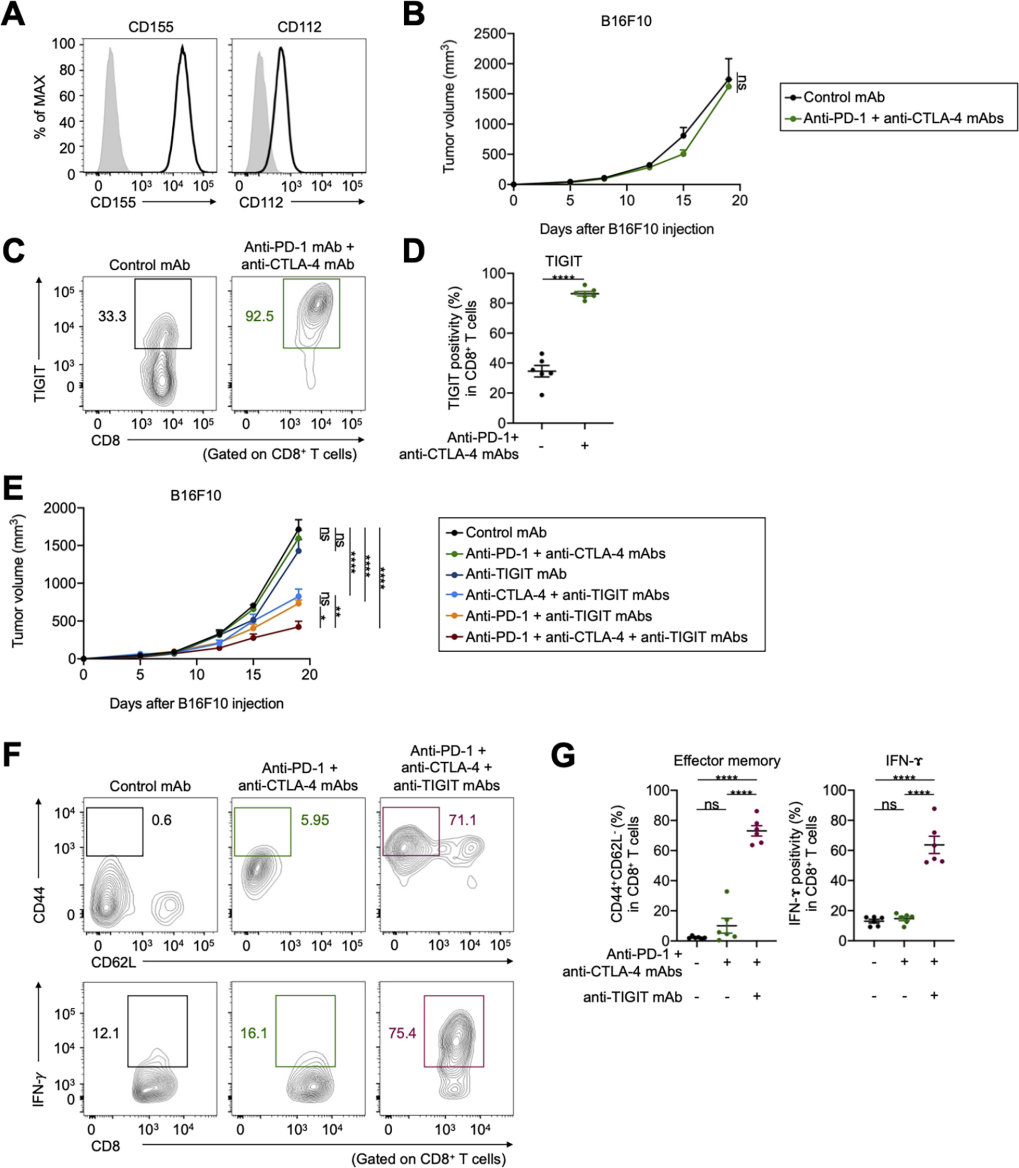
TIGIT blockade overcomes resistance to ICIs in vivo mouse models. (A) CD155 and CD112 expression in B16F10 cells. Representative flow cytometry staining from triplicated experiments are shown. Gray, isotype. (B) Tumor growth treated with combination treatment of anti-PD-1 and anti-CTLA-4 mAbs. Cells (1×10^6^) were injected subcutaneously (n=5 per each group), and tumor volume was monitored two times a week. Mice were grouped when the tumor volume reached approximately ~100 mm^3^, and ICIs were administered intraperitoneally three times every 3 days thereafter. (C and D) TIGIT expression in tumor-infiltrating CD8^+^ T cells. Tumors were harvested 7 days after treatment initiation to collect TILs for evaluation. Representative flow cytometry staining (C) and summary (D) are shown. (E) Tumor growth treated with combination treatment of anti-PD-1, anti-CTLA-4, and anti-TIGIT mAbs. In vivo experiments were performed as described in (B). (F and G) The frequencies of CD44^+^CD62L^−^ effector memory, and cytokine-producing CD8^+^ T cells in the TME. TILs were analyzed as described in (C and D). Representative flow cytometry staining (F) and summaries (G) are shown. All in vivo experiments were performed in duplicates, with similar results. A two-way ANOVA was used in (B), a t-test was used in (D), two-way ANOVA with Bonferroni corrections were used in (E), and one-way ANOVA with Bonferroni corrections were used in (G) for statistical analyses. The means and SEM are shown. *, p<0.05; **, p<0.01; ****, p<0.0001; ANOVA, analysis of variance; CTLA-4, cytotoxic T-lymphocyte-associated protein 4; ICIs, immune checkpoint inhibitors; ns, not significant; PD-1, programmed death 1; TILs, tumor-infiltrating lymphocytes; TME, tumor microenvironment.

We performed in vivo mouse experiments using CD155-expressing mouse cell lines; furthermore, expression of another TIGIT ligand, CD112, in these cell lines was comparable (figure 4A and online supplemental figure S4A). These tumors were resistant to combination with anti-PD-1 and anti-CTLA-4 mAbs (figure 4B and online supplemental figure S4B). TIGIT was highly expressed in tumor-infiltrating CD8^+^ T cells from treated mice compared with that in the control (figure 4C, D). Addition of anti-TIGIT mAb aided in overcoming the resistance (figure 4E and online supplemental figure S4B). Furthermore, the addition of TIGIT blockade to PD-1 or CTLA-4 blockade partially overcame the resistance (figure 4E and Supplementary Figure S4B). In contrast, the antitumor effect of ICIs was not observed in B6 SCID immunodeficient mice (online supplemental figure S4C). Accordingly, addition of anti-TIGIT mAb increased the proportion of effector memory CD8^+^ T cells, as well as cytokine production in tumor-infiltrating CD8^+^ T cells (figure 4F, G).

**Figure 5:**
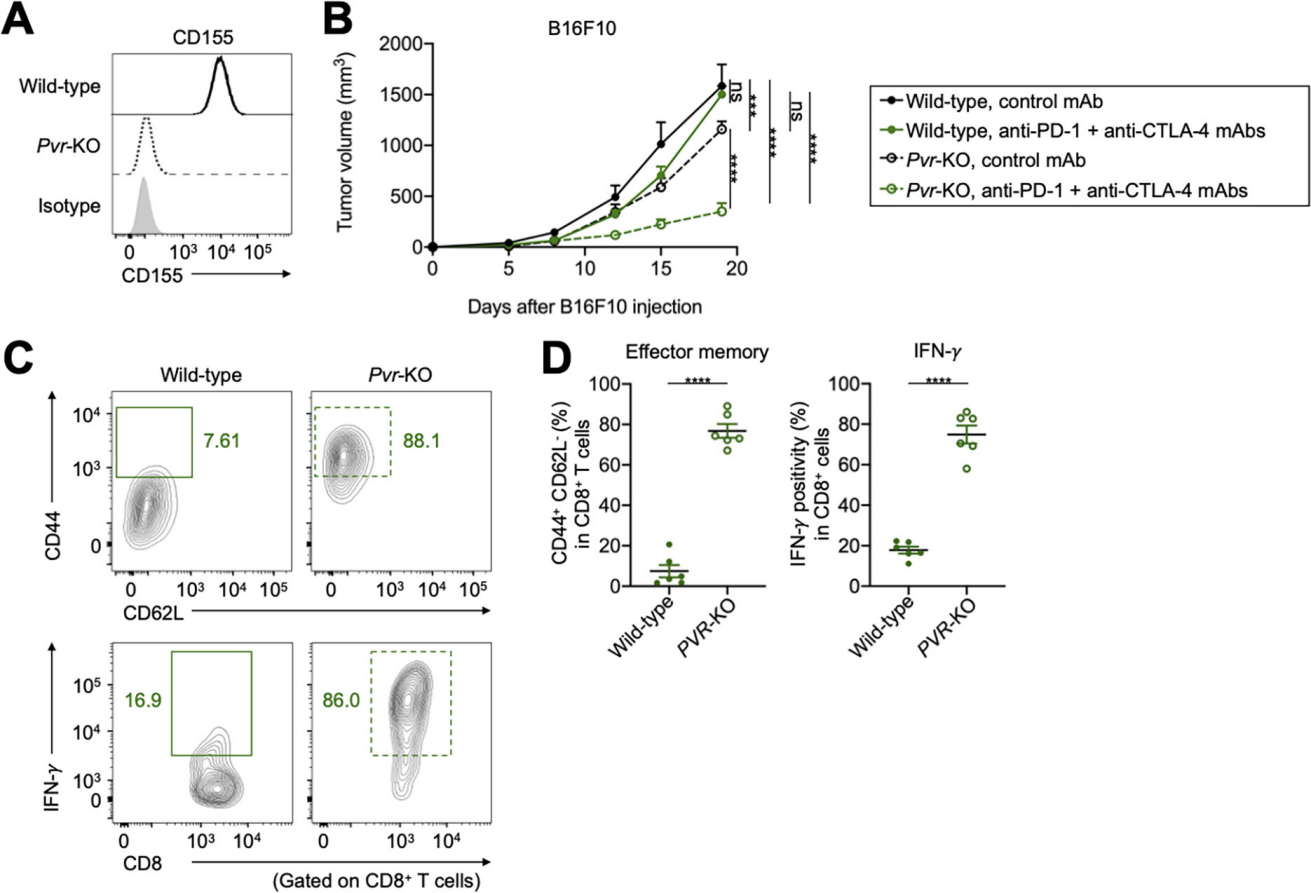
CD155-deletion overcomes resistance to ICIs in vivo mouse model. (A) CD155 expression in *pvr*-KO B16F10 cells. Representative flow cytometry staining from triplicated experiments are shown. (B) Tumor growth treated with combination treatment of anti-PD-1 and anti-CTLA-4 mAbs. Cells (1×10^6^) were injected subcutaneously (n=5 per each group), and tumor volume was monitored two times a week. Mice were grouped when the tumor volume reached approximately ~100 mm^3^, and ICIs were administered intraperitoneally three times every 3 days thereafter. (C and D) The frequencies of CD44^+^CD62L^−^ effector memory, and cytokine-producing CD8^+^ T cells in the tumor microenvironment treated with combination treatment of anti-PD-1 and anti-CTLA-4 mAbs. Tumors were harvested 7 days after treatment initiation to collect tumor-infiltrating lymphocytes for evaluation. Representative flow cytometry staining (C) and summaries (D) are shown. All in vivo experiments were performed in duplicates, with similar results. Two-way analysis of variance with Bonferroni corrections were used in (B), and t-tests were used in (D) for statistical analyses. The means and SEM are shown. ***, p<0.001; ****, p<0.0001; CTLA-4, cytotoxic T-lymphocyte-associated protein 4; ns, not significant; PD-1, programmed death 1.

Next, *pvr*-KO B16F10 and EMT6 cell lines were created using CRISPR/Cas9 technology (figure 5A and online supplemental figure S4D), and similar in vivo experiments were performed. *Pvr*-KO tumors grew slightly slower than wild-type tumors, and combination with anti-PD-1 and anti-CTLA-4 mAbs inhibited *pvr*-KO tumor growth (figure 5B and online supplemental figure S4E). In contrast, there was no significant difference in tumor growth between wild-type and *pvr*-KO tumors, and antitumor efficacy of ICIs was not observed in B6 SCID immunodeficient mice (online supplemental figure S4F). Consistently, the proportion of effector memory CD8^+^ T cells, as well as cytokine production in tumor-infiltrating CD8^+^ T cells increased with *pvr*-deletion (figure 5C, D). These findings indicate that the TIGIT/CD155 axis mediates resistance to ICIs in in vivo mouse models, which can be overcome by TIGIT/CD155 blockade.

### CD155 contributes to primary and acquired resistance to ICIs in patients with melanoma with inflamed TME

**Figure 6:**
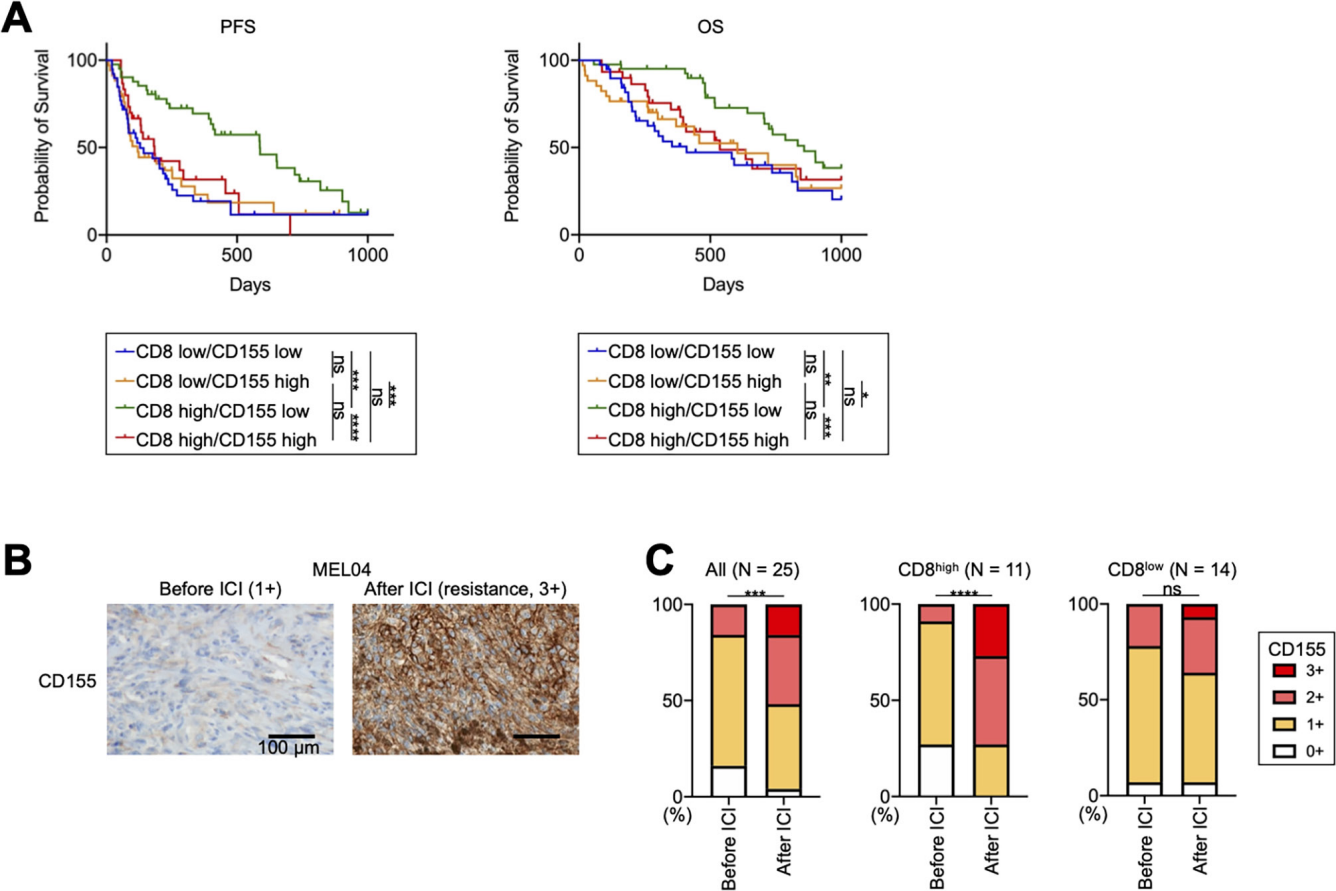
CD155 expression is related to resistance to ICIs in patients with melanoma with inflamed tumor microenvironment, including acquired resistance. (A) Survival curves of patients with melanoma. We analyzed a prognosis in 144 patients with melanoma who received ICIs. PFS and OS were defined as the time from the initiation of first ICIs until the first observation of disease progression or death from any cause and the time from the initiation of first ICIs until death from any cause, respectively. Survival curves according to CD8^+^ T-cell infiltration and CD155 expression are shown. (B and C) CD155 staining in MEL04 and CD155 expression change from baseline to resistance state. We stained paired formalin-fixed, paraffin-embedded samples at both baseline (before ICI) and resistance state (after ICI) in 25 patients with melanoma who received ICIs, and evaluated their staining as scores (0, 1+, 2+, and 3+). In addition to all 25 patients, the CD155 expression change was also analyzed according to CD8^+^ T-cell infiltration at resistance state. CD155 staining in MEL04 (B) and the summary of changes (C) are shown. Survival curves were analyzed using the Kaplan-Meier method and compared among groups using the log-rank test in (A), and paired t-tests were used in (C) for statistical analyses. *, p<0.05; **, p<0.01; ***, p<0.001; ****, p<0.0001; ICIs, immune checkpoint inhibitors; ns, not significant; OS, overall survival; PFS, progression-free survival.

We analyzed additional clinical samples from 144 patients with melanoma who received ICIs (online supplemental table S2). We included 13 patients who received ICIs as second-line therapy in our analyses (online supplemental table S2). They have received BRAF/MEK inhibitors prior to ICIs, and their pretreatment samples were obtained before treatment with BRAF/MEK inhibitors. The PFS of patients receiving ICIs as second-line therapy was significantly shorter than that of patients as first-line therapy, as previously reported^33^ (online supplemental figure S5A). In Japan, acral and mucosal types are dominant,^34^ and our study also included 81 patients with these types (online supplemental table S2). While acral and mucosal melanomas reportedly have a non-inflamed TME with less efficacy of ICIs,^35–37^ a Japanese prospective observational study has shown that anti-PD-1 mAb exhibits similar efficacy against these types.^34^ We analyzed our cohort according to the type and found that the OS of patients with acral or mucosal melanoma was slightly shorter than that of the others (online supplemental figure S5B), which is consistent with previous studies.^35–37^ Next, we stained the FFPE samples for CD8 and CD155 before ICI treatment (online supplemental figure S6A). We set a cut-off for CD8 count and CD155 score, which was <82.8/≥82.8 and 0–1/2–3, respectively, from the ROC curves using 6-month PFS (online supplemental figure S6B). There was no significant difference in CD8^+^ T-cell infiltration and CD155 expression among melanoma types (online supplemental table S2). Patients with high CD8^+^ T-cell infiltration had significantly longer PFS and OS than those with low CD8^+^ T-cell infiltration, which is consistent with the results of previous reports (online supplemental figure S6C).^11 38 39^ Additionally, patients with high CD155 expression tended to have a worse prognosis, as previously reported (online supplemental figure S6D).^30^ We next divided these patients into four groups: CD8 low/CD155 low (n=39), CD8 low/CD155 high (n=34), CD8 high/CD155 low (n=41), and CD8 high/CD155 high (n=30). Patients with CD8 high/CD155 low had significantly longer PFS and OS from other patients, whereas patients with CD8 high/CD155 high had significantly shorter PFS and OS than those with CD8 high/CD155 low and comparable with those with CD8 low (figure 6A). This good prognosis group (CD8 high/CD155 low) included 19 cutaneous or primary unknown and 22 acral or mucosal melanomas, respectively (19/63 vs 22/81, p=0.69). Consistently, the multivariate analyses demonstrated that immune status (CD8 high/CD155 low vs others), but not either treatment line or melanoma type, was an independent prognostic factor in our cohort (online supplemental table S6).

We also obtained both pretreatment and post-treatment paired samples from 25 patients who developed resistance after ICI treatment (online supplemental table S3). Thus, all samples after the ICIs were obtained in the resistance state. CD155 expression in resistant tumor cells significantly increased from the baseline as was observed in MEL04 (figure 6B, C). CD155 expression was particularly increased in patients with high CD8^+^ T cell infiltration at the resistance state (≥82.8) (figure 6C), whereas it was comparable in other patients (<82.8). Altogether, the TIGIT/CD155 axis induces resistance to ICIs with the inflamed TME, including both primary and acquired resistance (‘inflamed resistance’).

## Discussion

ICIs such as anti-PD-1 mAb and anti-CTLA-4 mAb provide clinical benefits in many types of cancer including melanoma and lung cancer.^5–7^ However, many patients fail to respond primarily, and others acquire resistance after initial response, necessitating further understanding of the resistance mechanisms.^18 19^ Neoantigens derived from somatic mutations induce a strong immune response, leading to an inflamed TME.^10 15 16^ ICIs can reinvigorate effector T cells in the inflamed TME, resulting in tumor regression.^10–14^ Thus, inflamed TME and high TMB are representative predictive biomarkers.^10–16^ However, MEL04 involved an inflamed TME and extremely high TMB but was resistant to ICIs. Thus, we focused on immune suppressive factors, especially co-inhibitory molecules. From RNA-seq data, we observed that CD155, a TIGIT ligand, was highly expressed in the MEL04 cell line, compared with other ligands. TIGIT was also highly expressed in MEL04 tumor-infiltrating T cells compared with other co-inhibitory checkpoint molecules such as LAG-3 and TIM-3. Accordingly, in vitro assays using clinical samples, and in vivo mouse models showed that the TIGIT/CD155 axis could mediate resistance to ICIs. Altogether, MEL04 was resistant to ICIs due to the TIGIT/CD155 axis despite having inflamed TME and extremely high TMB. In addition, we report for the first time that the TIGIT/CD155 axis can contribute to such inflamed resistance, including both primary and acquired resistance, in another large melanoma cohort. Considering the contribution of the TIGIT/CD155 axis to inflamed resistance, TIGIT blockade could be a potential candidate for combination treatment against inflamed resistant patients.

CD155 is an adhesion molecule expressed in tumor cells and tumor-associated myeloid cells that functions as a co-inhibitory molecule.^24 25^ CD155 interacts with a co-stimulatory molecule, CD226, and co-inhibitory molecules, TIGIT and CD96, resulting in either immune-cell activation or inhibition, respectively.^25 26^ Although TIGIT also binds to CD112, it exhibits the strongest affinity for CD155.^25 26^ Thus, the TIGIT/CD155 axis reportedly mediates immunosuppression in various immunological fields, including cancer immunology,^40–44^ therapeutic targeting of the TIGIT/CD155 axis has demonstrated efficacy and synergistic activity with anti-PD-1/PD-L1 mAb in preclinical tumor models,^45 46^ and high expression of CD155 in tumor cells is associated with decreased sensitivity to ICIs in clinical samples.^30^ In addition, several studies have indicated the clinical significance of TIGIT, including poor prognosis and resistance to ICIs.^46–48^ Compared with these studies, our study is valuable in that we validated the inflamed resistance using a considerable number of clinical samples, including both primary and acquired resistance, and its mechanisms using pairs of autologous tumor cell lines and TILs in addition to in vivo mouse models. Furthermore, we created an acquired resistance model using human clinical samples. In vivo mouse models, LAG-3 and TIM-3 are related to acquired resistance.^22^ Our model, using human clinical samples, clearly demonstrated that the TIGIT/CD155 axis induces acquired resistance to ICIs, in particular inflamed resistance, which was observed in MEL04 and another large melanoma cohort. That is, TIGIT expression in effector T cells increased via TCR signaling pathways, which can be activated by PD-1 blockade,^32^ in the inflamed TME, and such TIGIT^+^ effector T cells were subsequently suppressed by CD155 expressed in tumor cells, leading to the survival of CD155-expressing tumor cells and resistance. In contrast, CD155 is reportedly regulated by nuclear factor-κB (NFκB) signaling pathways.^49^ Thus, such an inflammatory response in the TME could also induce CD155-mediated resistance. In addition, TIGIT is reportedly a chronic-stimulated exhausted T cell marker, in addition to PD-1.^8 9^ Accordingly, high expression of TIGIT can also be related to resistance to ICIs, such as CD155.^30 47 48^ Furthermore, the expression in effector T cells and regulatory T cells reportedly induces resistance.^47 48^ From these findings, chronic stimulation can drive increased expression of TIGIT and CD155 and/or selection for cells expressing these proteins. Further research is warranted to elucidate the detailed mechanisms of resistance.

This study has some limitations. The number of human clinical samples in the functional assays was small because it is difficult to establish tumor cell lines, and both two resistance samples were obtained at acquired resistance. Thus, to generalize our findings, we performed in vivo mouse experiments. Furthermore, we validated the contribution of the TIGIT/CD155 axis to resistance in another large melanoma cohort, showing that the TIGIT/CD155 axis could induce inflamed resistance, including both primary and acquired resistance. While further research is warranted, we believe that the TIGIT/CD155 axis can generally induce inflamed resistance, including both primary and acquired resistance. In addition, these findings can be expanded beyond melanoma to other cancer types, such as lung cancer, although we analyzed only melanoma samples. Indeed, a randomized phase 2 trial of combination therapy with anti-PD-L1 mAb and anti-TIGIT mAb has demonstrated favorable efficacy against PD-L1 high lung cancer, which can reflect inflamed TME,^50^ and a phase 3 trial is currently ongoing (NCT04294810). Acral and mucosal melanomas are dominant in Japan,^34^ and our cohort included a considerable number of these types. While acral and mucosal melanomas reportedly have a non-inflamed TME with less efficacy of ICIs,^35–37^ a Japanese prospective observational study has shown that anti-PD-1 mAb exhibits similar efficacy against these types.^34^ In our cohort, there was no significant difference in CD8^+^ T-cell infiltration or CD155 expression among melanoma types. While patients with acral or mucosal melanoma had a slightly shorter OS, multivariate analyses demonstrated that the melanoma type was not a prognostic factor. While larger research is required, it seems challenging to consider whether the melanoma type can reflect the immune status from our analyses.

In summary, we have shown that the TIGIT/CD155 axis contributes to resistance to ICIs in both in vitro functional assays using clinical samples and in vivo mouse models. CD155 was highly expressed in tumor cells from resistant patients, including both primary and acquired resistance, despite the inflame TME from another large melanoma cohort. TIGIT blockade therapies could be candidates for combination treatment against inflamed resistant patients. We propose the development of combination therapies with TIGIT blockade against resistant patients with inflamed TME and high TIGIT/CD155 expression.

10.1136/jitc-2021-003134.supp2 Supplementary data

## Data Availability

Data are available upon reasonable request. The data that support the findings of this study are available from the corresponding author, YT, upon reasonable request. Whole exome sequencing data and RNA-seq data are deposited in JGAS000285.
