# Supplementary material for: TIGIT/CD155 axis mediates resistance to immunotherapy in patients with melanoma with the inflamed tumor microenvironment
Source: J Immunother Cancer. 2021 May 10;9(11):e003134. doi: 10.1136/jitc-2021-003134 (PMC8603290; doi:10.1136/jitc-2021-003134)
Supplement: Supplementary data [file jitc-2021-003134supp001.pdf]

Supplementary Figure S1. Clinical courses of MEL04 and additional analyses of tumor cell lines

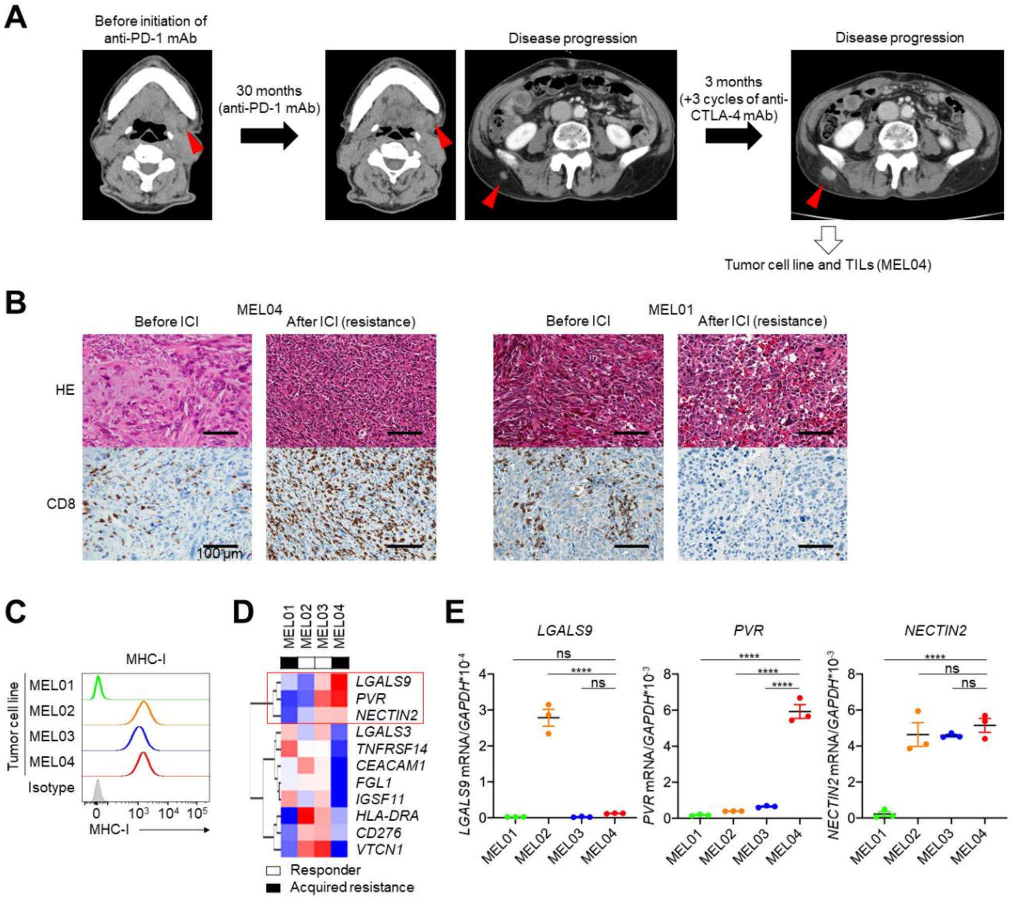

(A) Computed tomography (CT) imaging of MEL04. CT scan before initiation of anti-PD-1 mAb, at disease progression after therapy, and at disease progression after adding anti-CTLA-4 mAb are shown. Red arrows, lymph node metastases. **(B)** Immunohistochemistry. The surgical resected samples from MEL04 before initiation of anti-PD-1 mAb and at acquired resistance were stained for HE (top) and CD8 (bottom). As control, we stained the MEL01 samples. **(C)** MHC-I expression in tumor cells. MHC-I expression was analyzed with flow cytometry. Representative flow cytometry staining is shown from triplicate experiments. **(D)** Heatmap of immune suppressive molecules in tumor cells. Unsupervised clustering was performed using RNA-seq data. **(E)** LGAL9 (left), PVR (middle), and NECTIN2 (right) gene expression in tumor cells. Total RNA was reversed to cDNA and real-time PCR was performed. *GAPDH* was used as internal control.

All *in vitro* experiments were performed in triplicate, and one-way ANOVA with Bonferroni corrections were used in (E) for statistical analyses. The means and SEM are shown. ****, $P < 0.0001$; HE, Hematoxylin Eosin; ns, not significant.

Supplementary Figure S2. TIGIT expression in peripheral blood T cells from healthy donors

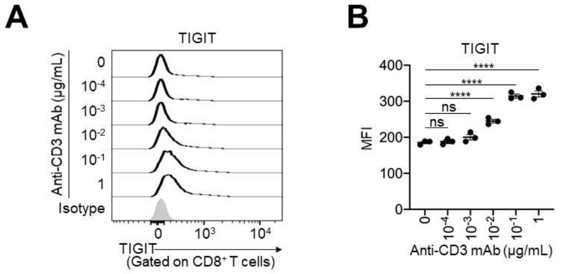

PBMCs from healthy donors were stimulated by anti-CD3 mAb at the indicated concentrations and anti-CD28 mAb for 48 hours, and TIGIT expression was subsequently analyzed with flow cytometry. Representative flow cytometry staining from triplicate experiments (A) and summary of MFI (B) are shown.

All *in vitro* experiments were performed in triplicate, and one-way ANOVA with Bonferroni corrections were used in (B) for statistical analyses. The means and SEM are shown. ****, $P < 0.0001$; ns, not significant; MFI, mean fluorescent intensity.

Supplementary Figure S3. CD155 expression from *in vitro* acquired resistance model for one month

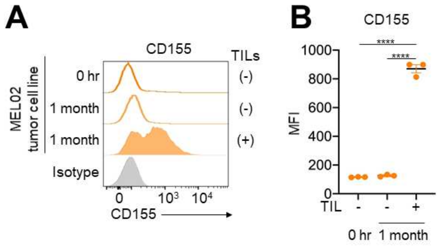

Autologous tumor cells were cocultured with exchanging paired TILs every week for one month, and tumor cells were subsequently analyzed by flow cytometry. Representative flow cytometry staining (A) and summary (B) are shown.

All *in vitro* experiments were performed in triplicate, and one-way ANOVA with Bonferroni correction was used in (B) for statistical analyses. The means and SEM are shown. ****, $P < 0.0001$; MFI, mean fluorescent intensity.

Supplementary Figure S4. *In vivo* experiments of EMT6 tumors in wild-type mice and B16F10 in SCID immunodeficient mice

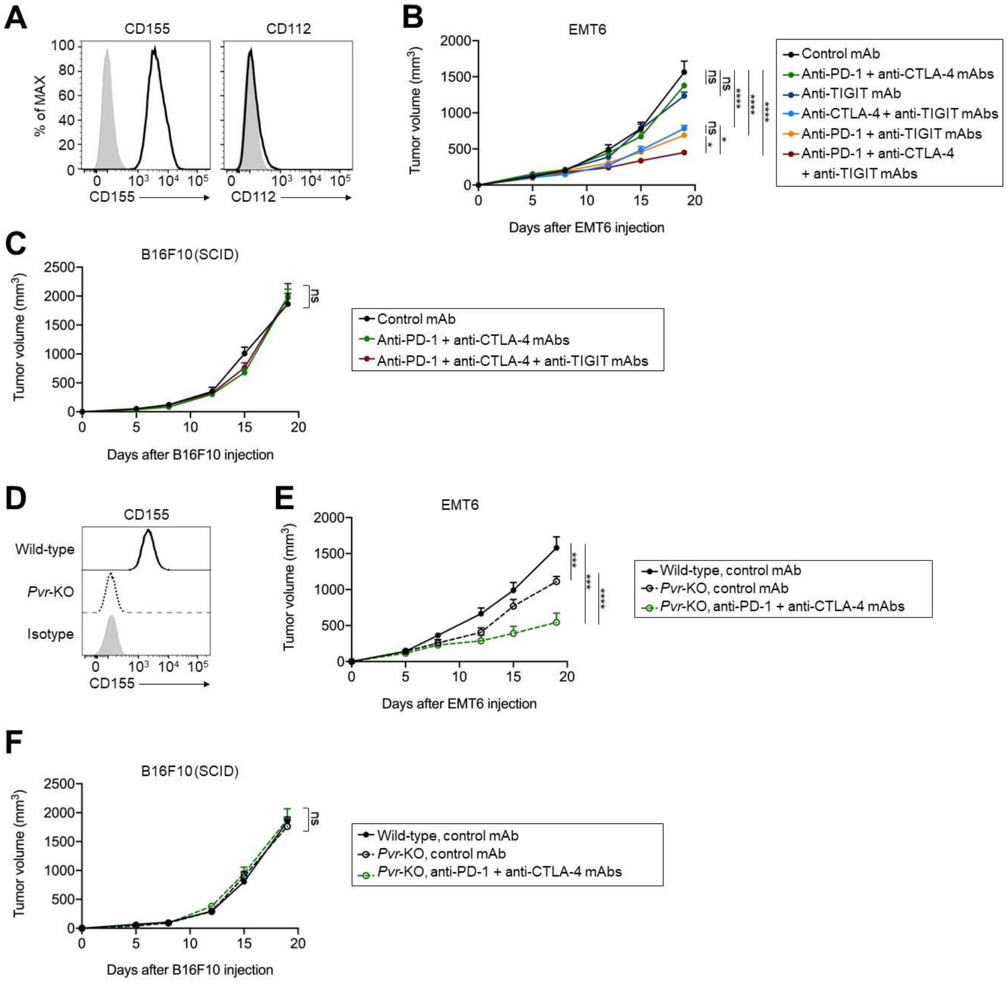

(A) CD155 and CD112 expression in EMT6 cells. Representative flow cytometry staining from triplicate *in vitro* experiments are shown. Gray, isotype. **(B)** Tumor growth of EMT6 treated with combination treatment of anti-PD-1, anti-CTLA-4, and anti-TIGIT mAbs in wild-type mice. Cells (1×10^6) were injected subcutaneously (n = 5 per each group), and tumor volume was monitored twice a week. Mice were grouped when the tumor volume reached approximately ~100 mm^3, and ICIs were administered intraperitoneally three times every three days thereafter. **(C)** Tumor growth of wild-type B16F10 treated with combination treatment of anti-PD-1, anti-CTLA-4, and anti-TIGIT mAbs in SCID mice. *In vivo* experiments were performed as described in (B) **(D)** CD155 expression in *pvr*-KO EMT6 cells. Representative flow cytometry staining from triplicate *in vitro* experiments are shown. **(E)** Tumor growth of EMT6 treated with combination treatment of anti-PD-1 and anti-CTLA-4 mAbs in wild-type mice. *In vivo* experiments were performed as described in (B). **(F)** Tumor growth of *pvr*-KO B16F10 treated with combination treatment of anti-PD-1 and anti-CTLA-4 mAbs in SCID mice. *In vivo* experiments were performed as described in (B).

All *in vivo* experiments were performed in duplicates with similar results, two-way ANOVA with Bonferroni corrections were used in (B) (C) (E) and (F) for statistical analyses. The means and SEM are shown. *, $P < 0.05$; ***, $P < 0.001$; ****, $P < 0.0001$; ns, not significant.

Supplementary Figure S5. Survival curves according to treatment line or melanoma type

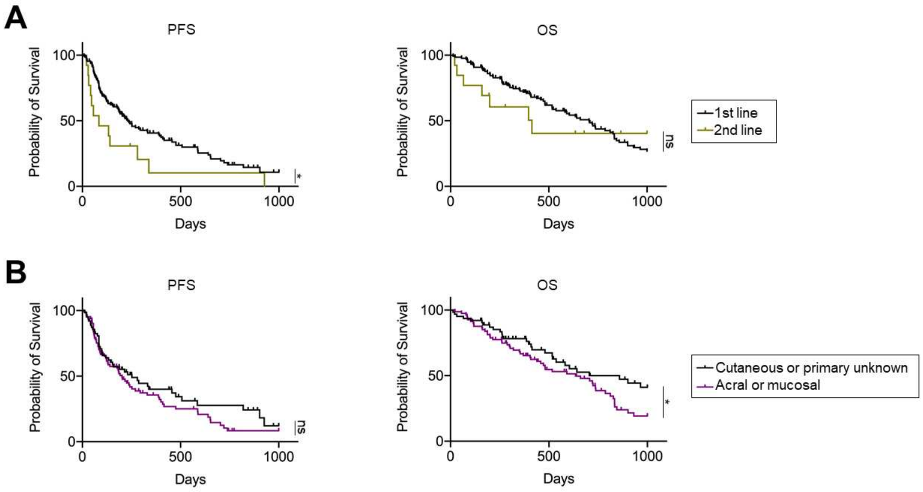

We analyzed the prognosis of 144 melanoma patients who received ICIs. PFS and OS were defined as the time from the initiation of the first ICIs until the first observation of disease progression or death from any cause and the time from the initiation of the first ICIs until death from any cause, respectively. Survival curves according to treatment line (A) or melanoma type (B) are shown.

The Kaplan–Meier methods and log-rank tests were used for statistical analyses. *, $P < 0.05$; ns, not significant.

Supplementary Figure S6. IHC for CD155 and CD8, and survival curves

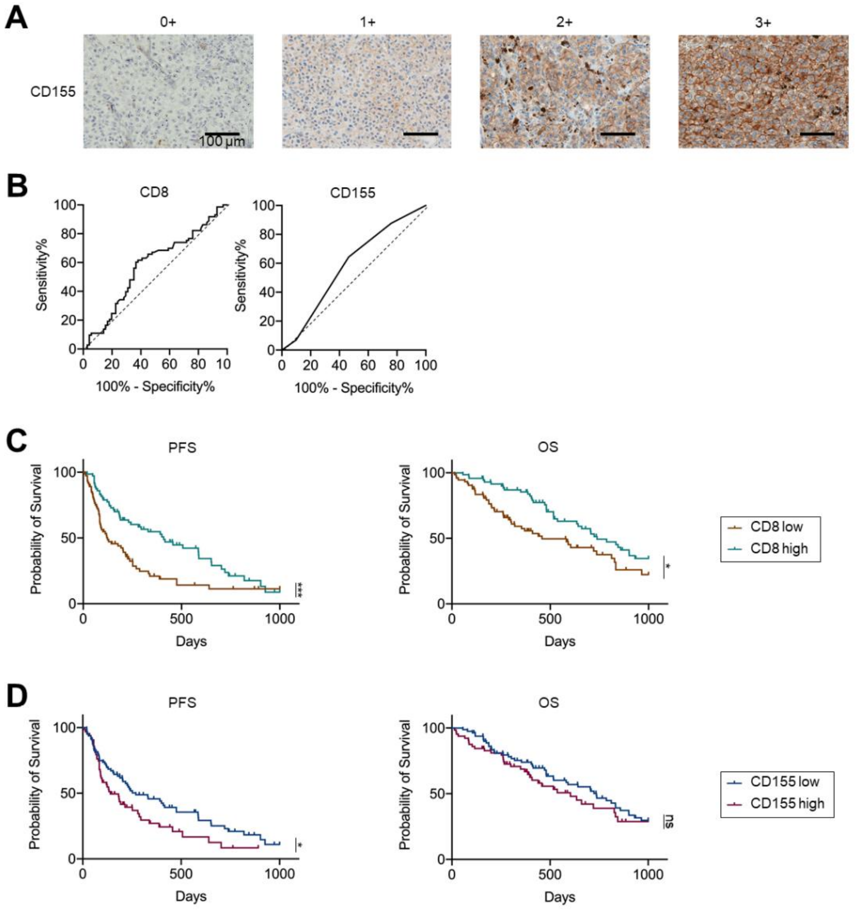

(A) Representative staining figures. We stained FFPE samples for CD155 at baseline (before ICI) in 144 melanoma patients who received ICIs, and evaluated their staining as scores (0, 1+, 2+, and 3+). Scale bar, 100 µm. **(B)** ROC curves for CD8 count and CD155 score. We also stained FFPE samples for CD8 using the same cohort, and intratumoral $CD8^+$ T cells were counted: 5 fields (0.25 mm^2) were randomly selected and counted from each slide. Six-month PFS was used to draw the ROC curves and set cutoffs. **(C** and **D)** Survival curves of melanoma patients. We analyzed the prognosis of 144 melanoma patients who received ICIs. PFS and OS were defined as the time from the initiation of the first ICIs until the first observation of disease progression or death from any cause and the time from the initiation of the first ICIs until death from any cause, respectively. Survival curves according to $CD8^+$ T-cell infiltration (C) or CD155 expression (D) are shown.

The Kaplan–Meier methods and log-rank tests were used in (C) and (D) for statistical analyses, respectively. *, $P < 0.05$; ***, $P < 0.001$; ns, not significant.

Supplementary Table S1. Patient characteristics of MEL01-04

Case	Age	Sex	Type	Stage	BRAF	PD-1 blockade		Sampling
						**Response**	**PFS**	
**MEL01**	Middle 50s	F	Acral	T4aN1a M0	Wild type	PR→PD	18 months	After PD-1 blockade, acquired resistant
**MEL02**	Middle 80s	M	Acral	TxN3 M0	Wild type	CR continue	More than 2 years	Before PD-1 blockade
**MEL03**	Late 60s	M	Cutaneous	T4bN2 M0	Wild type	CR continue	More than 2 years	Before PD-1 blockade
**MEL04**	Early 70s	M	Cutaneous	T4bN2 M1b	Wild type	PR→PD	30 months	After PD-1 and CTLA-4 blockade, acquired resistant

F, female; M, male; PD, progressive disease; PR, partial response; CR, complete response; PFS, progression-free survival.

Supplementary Table S2. Patient characteristics for immunohistochemistry

**Features**	**CD8**			**CD155**		
	**Low (n = 73)**	**High (n = 71)**	***P***	**Low (n = 80)**	**High (n = 64)**	***P***
**Age, years** [median] (range)	69 (21–89)	70 (25–87)	0.64	70 (22–86)	69.5 (21–89)	0.64
**Sex** (male/female)	45/28	38/33	0.40	42/38	41/23	0.18
**Performance status** (0 or 1/2 or 3)	68/5	69/2	0.44	77/3	60/4	0.70
**Type** (cutaneous/primary unknown/acral/mucosal)	21/7/30/15	28/7/28/8	0.24¶	26/4/34/16	23/10/24/7	0.095¶
**Stage** (I-III/IV)	25/48	26/45	0.86	26/54	24/40	0.60
**ICI** (anti-PD-1 mAb/anti-CTLA-4 mAb/combination)	53/3/17	61/4/6	0.022¶¶	61/6/13	53/1/10	>0.99¶¶
**ICI treatment line** (1st line/2nd line)	67/6	64/7	0.78	77/3	54/10	0.018
***BRAF* status** (mutated/wild-type/NE)	14/55/4	18/47/6	0.42	13/60/7	19/42/3	0.10
**Serum LDH** [median] (range)	207 (106–2194)	208 (81–982)	0.97	206.5 (106–1185)	207 (81–2194)	0.19
**CD155** (0–1/2–3)	39/34	41/30	0.62			
**CD8** [median] (range)				82.1 (2-610)	71.9 (1.8-338.8)	0.62
**Response to ICIs** (responder/non-responder¶¶¶)	28/45	45/26	0.0009	47/33	26/38	0.12

NE, not evaluated. ¶cutaneous or primary unknown vs. acral or mucosal ¶¶Anti-PD-1 mAb or anti-CTLA-4 mAb vs. combination therapy ¶¶¶Responders were defined as patients with a PFS ≥6 months.

Supplementary Table S3. Clinical features of patients who provided paired samples

**Features**	**n = 25**		
**Age, years** [median] (range)	63 (38–84)		
**Sex** (male/female)	12/13		
**Performance status** (0-1/2-)	24/1		
**Type**<br>(cutaneous/primary unknown/acral/mucosal)	10/0/10/5		
**Stage** (I–III/IV)	14/11		
**ICI**<br>(anti-PD-1 mAb/anti-CTLA-4 mAb/combination)	23/0/2		
**ICI treatment line**<br>(1st line/2nd line)	23/2		
***BRAF* status**<br>(mutated/wild-type/NE)	6/18/1		
**Serum LDH** [median] (range)	191 (143–332)		
**CD155** (0–1/2–3)	**Before ICI**	**After ICI**	***P***
	21/4	13/12	0.032
**CD8** [median] (range)	**Before ICI**	**After ICI**	***P***
	82.2<br>(4-336)	115.2<br>(17-485)	0.25

NE, not evaluated.

Supplementary Table S4. Primers for real-time PCR

Gene	Sequence	
	Forward	Reverse
LGALS9	TCAAGGAGGTCTCCAGGACG	TGAAAGTTCACAGCAAACCT GG
PVR	CACTCAGGCATGTCCCGTAA	CATGCTCTGTACTCGAGGGA
NECTIN2	CGGAACTGTCACTGTCACCA	GACACTTCAGGAGGGTAGCG
GAPDH	ACCACAGTCCATGCCATCAC	TACAGCAACAGGGTGGTGGA

PCR, polymerase chain reaction

Supplementary Table S5. Summary of the antibodies used in flow cytometry analyses

Molecule	Clone	Tag	Company
**Human CD3**	SK7	PE-Cy7	BioLegend
**Human CD8**	SK1	FITC	BD Biosciences
**Human TIGIT**	MBSA43	PE	Thermo Fisher Science
**Human LAG-3**	3DS223H	PE	Thermo Fisher Science
**Human TIM-3**	F38-2E2	PE	Thermo Fisher Science
**Human CD155**	2H7CD155	APC	Thermo Fisher Science
**Human CD112**	TX31	PE	BioLegend
**Human MHC-II**	Tü; 39	PE-Cy7	BioLegend
**Human Galectin-3**	M3/38	APC	BioLegend
**Human Galectin-9**	9M1-3	PerCP-Cy5.5	BioLegend
**Human MHC-I**	W6/32	FITC	Thermo Fisher Science
**Mouse CD155**	TX56	APC	BioLegend
**Mouse CD112**	829038	PE	R&D systems
**Mouse CD3**	500A2	V500	BD Biosciences
**Mouse CD4**	RM4-5	PE-Cy7	BD Biosciences
**Mouse CD8**	53-6.7	PerCP-Cy5.5	BD Biosciences
**Mouse PD-1**	29F.1A12	V450	BioLegend
**Mouse TIGIT**	1G9	PE	BioLegend
**Mouse CD44**	IM7	APC	BioLegend
**Mouse CD62L**	MEL-14	PE	BioLegend
**Mouse IFN-γ**	1.2	APC	BD Biosciences

Supplementary Table S6. Multivariate analyses

**Features**	**PFS**			**OS**		
	**HR**	**95% CI**	***P***	**HR**	**95% CI**	***P***
**Age, years**<br>(< 70 vs. ≥ 70)	1.06	0.66-1.72	0.81	0.86	0.49-1.50	0.60
**Sex**<br>(female vs. male)	1.26	0.78-2.03	0.35	1.24	0.71-2.18	0.46
**Performance status**<br>(0 or 1 vs. 2 or 3)	0.57	0.21-1.52	0.26	0.18	0.067-0.50	0.0010
**Type**<br>(cutaneous or primary unknown vs. acral or mucosal)	0.78	0.50-1.27	0.32	0.80	0.44-1.43	0.45
**Stage**<br>(I-III or IV)	0.42	0.26-0.69	0.0006	0.29	0.16-0.54	<0.0001
**ICI**<br>(combination vs. monotherapy)	0.77	0.29-2.02	0.60	1.15	0.42-3.13	0.79
**ICI treatment line**<br>(1st line vs. 2nd line)	0.59	0.25-1.40	0.23	0.50	0.16-1.55	0.23
***BRAF* status**<br>(mutated vs. wild-type)	1.25	0.61-2.56	0.54	0.44	0.17-1.14	0.091
**Serum LDH**<br>(≤ upper limit vs. > upper limit)	0.69	0.43-1.10	0.12	0.41	0.24-0.69	0.0008
**Immune status**<br>(CD8 high/CD155 low vs. others)	0.32	0.19-0.54	<0.0001	0.34	0.19-0.62	0.0004
