# Supplementary material for: TIGIT/CD155 axis mediates resistance to immunotherapy in patients with melanoma with the inflamed tumor microenvironment
Source: J Immunother Cancer. 2021 May 10;9(11):e003134. doi: 10.1136/jitc-2021-003134 (PMC8603290; doi:10.1136/jitc-2021-003134)
Supplement: Supplementary data [file jitc-2021-003134supp002.pdf]

TIGIT/CD155 axis mediates resistance to immunotherapy in melanoma patients with the inflamed tumor microenvironment

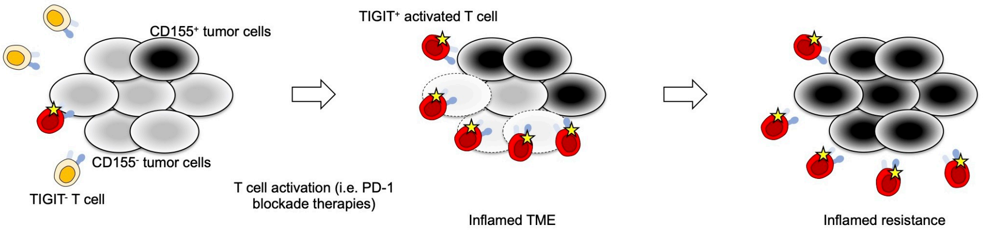

Authors
Shusuke Kawashima, Takashi Inozume, Masahito Kawazu, Toshihide Ueno, Joji Nagasaki, Etsuko Tanji, Akiko Honobe, Takehiro Ohnuma, Tatsuyoshi Kawamura, Yoshiyasu Umeda, Yasuhiro Nakamura, Tomonori Kawasaki, Yukiko Kiniwa, Osamu Yamasaki, Satoshi Fukushima, Yuzuru Ikehara, Hiroyuki Mano, Yutaka Suzuki, Hiroyoshi Nishikawa, Hiroyuki Matsue, and Yosuke Togashi

Correspondence
ytogashi1584@gmail.com

In Brief
TIGIT expression in activated effector T cells increases in the inflamed tumor microenvironment (TME), which are subsequently suppressed by CD155 expressed in tumor cells, leading to the survival of CD155-expressing tumor cells.
